# Associations between Diabetes and Quality of Life among Breast Cancer Survivors

**DOI:** 10.1371/journal.pone.0157791

**Published:** 2016-06-22

**Authors:** Zheng Tang, Jiwei Wang, Hao Zhang, Li Sun, Furong Tang, Qinglong Deng, Jinming Yu

**Affiliations:** 1 Institute of Clinical Epidemiology, Key Laboratory of Public Health Safety, Ministry of Education, School of Public Health, Fudan University, 130 Dong-An Road, Shanghai 200032, China; 2 Collaborative Innovation Center of Social Risks Governance in Health, Fudan University, Shanghai, China; 3 Division of Nephrology, Zhongshan Hospital, Shanghai Medical College, Fudan University, 180 Feng-Lin Road, Shanghai, China; 4 Shanghai Key Laboratory Kidney and Blood Purification, Shanghai 200032, China; School of Medicine, Fu Jen Catholic University, TAIWAN

## Abstract

**Objective:**

We aimed to investigate the associations between diabetes and quality of life (QOL) among breast cancer survivors.

**Methods:**

A cross-sectional survey was conducted at 34 Cancer Recovery Clubs across China from May 2014 to January 2015. Quality of life was measured by the Quality of Life Questionnaire-Core 30 (EORTC QLQ-C30) and the Quality of Life Questionnaire-Breast Cancer Module 23 (QLQ-BR23, simplified Chinese version). Information on social-demography, diagnosis and treatment of tumors, and diabetes mellitus were collected by self-reported questionnaires. Univariate analyses of covariance (ANCOVA) was performed to assess the difference in QOL between patients with or without diabetes mellitus, and multiple linear regression models were used to examine the associations after controlling for confounders.

**Results:**

Diabetes, both of type 1 diabetes (T1DM) and type 2 diabetes (T2DM) significantly reduced QOL. This effect of diabetes on QOL is independent of tumor size, regional lymph node metastasis, distant metastasis and tumor stage index (TNM). After adjusting for different social-demography, diagnosis and treatment of the tumor, the tumor’s stage and other chronic comorbidities, breast cancer survivors with diabetes got significantly lower scores in functional dimensions (including physical, role, emotional and social functionings measured by EORTC QLQ-C30; body image (BRBI) and future perspective (BRFU) measured by QLQ-BR23, as well as economic difficulties than those without diabetes (*P*_adjusted_<0.05). Diabetic patients also obtained higher scores in symptom dimensions, including fatigue, nausea and vomiting, pain, dyspnoea, insomnia, constipation and diarrhoea measured by EORTC QLQ-C30; side effects, breast symptoms and upset by hair loss measured by QLQ-BR23 (*P*_adjusted_<0.05). Compared to patients with T1DM, those with T2DM are likely to suffer more by loss of functioning.

**Conclusions:**

Diabetes was associated with the decreased QOL for breast cancer survivors.

## Introduction

Breast cancer has become a worldwide public health problem. In China, breast cancer is the most common tumor for women, and 169 thousand females were diagnosed with breast cancer every year [1]. Meanwhile, about 45 thousand females died of breast cancer [1]. In 2010, American Diabetes Association (ADA) and the American Cancer Society (ACS) jointly agreed that diabetic patients had a higher risk of getting breast cancer [2]. Moreover, diabetes is one of the most common comorbidities of breast cancer, and around 18% of breast cancer patients have diabetes [3, 4], even though 30% of them are not diagnosed [5]. Diabetes can increase the breast cancer morbidity by 5%~20%, especially among younger patients. For instance, the relative risk (RR) is 1.37 for patients aged between 55–64 and 2.43 for those aged between 25–54 [6]. The death rate of female breast cancer patients with glycosylated hemoglobin (HbA1C) above 7% is two times higher than that of the patients with HbA1C less than 6.5% [7]. It was also reported that these patients with diabetes tend to have bigger tumors, regional lymph node metastasis, and distant metastasis [8].

Previous studies reported that the comorbidity of diabetes may be a risk factor influencing the quality of life (QOL) of patients with breast cancer. Diabetes can shorten the disease-free survival of breast cancer patients and increase the mortality of patients [9]. Moreover, the patients who have both diabetes and breast cancer are more sensitive to toxin and at increased risk of the adverse effects after they received chemotherapy [10]. Meanwhile, the patients with HbA1C above 7% have a shorter disease-free survival [7]. However, to the best of our knowledge, there were only a few studies investigating the influence of diabetes on QOL for patients with breast cancer. In the present study, we performed a large cross-sectional survey to explore the association between diabetes and QOL for breast cancer survivors.

Several studies find that diabetes is a factor that adversely affects the occurrence and prognosis of breast cancer [11–14]. Insulin is involved in the biological mechanism, and it can accelerate cell division and consequently promote cancer progression [15–17]. Polypeptide hormones (Leptin) can increase the disease risk of diabetes and breast cancer to some extent, and they have acceleration functions to the generation of diabetes and breast cancer [18, 19]. Fat can increase the risks of getting diabetes and breast cancer by promoting the formation of insulin resistance and activating related signal passages of insulin/IGF, besides, they are prognostic dangerous factors [20]. Therefore, whether combing diabetes, especially T2DM, should become one of the important factors to judge the quality of life among breast cancer survivors.

## Materials and Methods

### Study population

A total of 6188 female breast cancer survivors were recruited from affiliated groups of Cancer Recovery Clubs in 34 cities across China. These Clubs are non-governmental organizations aiming to improve health and QOL of patients with different kinds of cancer in China. Participants fitting the following criteria were included in this study: (1) being a primary breast cancer, (2) active treatment completed, (3) having reading ability, and (4) free from mental disorders. A written Informed Consent was obtained from every participant and the study protocols were reviewed and proved by the Ethic Committee of Public Health School of Fudan University (protocol number RB #2013-04-0450).

### Measurement of diabetes

Information on diabetes including T1DM and T2DM was collected by self-reported questionnaires and was confirmed by physicians in ≥level 2 hospitals and health records.

### Measurement of Quality of Life (QOL) and the global health status (QL)

The QOL was measured by the simplified Chinese version of the Quality of Life Questionnaire-Core 30(EORTC QLQ-C30) and the Quality of Life Questionnaire-Breast Cancer Module 23 (QLQ-BR23).

The EORTC QLQ-C30 consists of five functional dimensions, three symptom dimensions, a global health status (QL), and six signal items, totaling 30 items. With 23 items, QLQ-BR23 is composed of four functional dimensions and four symptom dimensions. The original scores of each dimension were transformed into standard scores with a range of 0~100. The standard scores of functional and general health dimensions positively represented patients’ QOL, meanwhile the scores of symptom dimension negatively represented QOL. Higher scores for the functional scales represent a higher level of functioning and higher scores for the symptoms represent a greater extent of symptoms. The global health status (QL) scale was used as the overall summary measure. A high score for the QL represents a high QOL. QL are 7-point questions with range = 6.The level of self-assessed QL helps in predicting survival, which is especially important among survivors to improve the QOL.

### Statistical analysis

Data were analyzed with the Statistical Package for the Social Sciences (SPSS) for Windows (Version19.0). Participants’ characteristics and QOL were presented as percentages for categorical variables and mean ± standard deviation for continuous variables. Differences in terms of age, body mass index (BMI), years since diagnosis as well as QOL were analyzed using unpaired t-tests. Univariate analyses of covariance (ANCOVA) and multiple linear regression models were used to examine the effects of diabetes on different domains of QOL after controlling for age, BMI, education, household income, tumor characteristics (tumor size, regional lymph node metastasis, distant metastasis), and breast cancer treatment history. Statistical inferences were two-sided and *P* <0.05 was considered as statistical significant.

## Results

### The basic characteristics of study participants

As described in Table 1, the average age of the female breast cancer survivors included in this study was 56.9±9.0 years, with 80.3% of them being over 50 years old. Their average BMI was 24.1±5.0 Kg/m^2^, and overweight or obese survivors accounted for 29.8%. Of these 6188 study participants, 614 (9.9%) survivors had been diagnosed with diabetes, 131 (3.7%) were T1DM and 483 (7.8%) were T2DM.

**Table 1:** Characteristics of 6188 women with breast cancer, by diabetes status.

Characteristics	Total	%	Global health status (QL)		*P* value
	(N = 6188)				
Age(years)					0.003
<40	184	3.0	67.9±20.9		
40-	1035	16.7	68.7±21.9		
50-	2807	45.4	67.4±22.9		
60-	1853	29.9	65.6±21.5		
70-	309	5.0	65.2±20.5		
Body Mass Index (BMI, Kg/m^2^)					0.002
<18.5	226	3.7	62.2±23.1		
18.5-	4126	66.6	67.1±21.8		
25-	1504	24.3	67.9±23.0		
-30-	332	5.4	65.2±22.0		
Marital status					0.001
Married/with partner	5534	89.4	67.3±22.0		
Divorced/widowed/separated/ single	654	10.6	64.2±22.7		
Education			0.121		
Middle school or vocational school	2525	40.8	66.4±22.5		
Junior college or above	3663	59.2	67.4±21.9		
Monthly personal income (RMB)					0.210
Under 2000	2729	44.1	66.8±22.9		
2000–4000	2797	45.2	66.8±21.7		
Over 4000	662	10.7	68.8±20.5		
Primary tumor diameter (T)				<0.001	
T0	448	7.3	70.3±25.3		
≤20mm(T1)	3377	54.6	67.6±21.8		
20mm<T≤50mm(T2)	1906	30.8	66.4±21.8		
>50mm(T3)	343	5.5	63.7±23.4		
Chest wall and skin (T4)	114	1.8	62.5±17.2		
Regional lymph node metastasis (N)					<0.001
N0	4581	74.0	67.7±21.9		
N1	1124	18.2	65.7±22.7		
N2	264	4.3	64.4±21.8		
N3	219	3.5	62.5±23.2		
Distant metastasis (M)					<0.001
M0	5797	93.7	67.5±22.0		
M1	391	6.3	59.7±22.9		
TNM staging					<0.001
Stage0	366	5.9	70.1±25.7		
Stage1	2701	43.6	68.0±21.6		
Stage2	2214	35.8	67.1±22.0		
Stage3	516	8.3	65.0±21.7		
Stage4	391	6.3	59.7±22.85		
Diabetes					0.002
No	5574	90.1	67.3±22.0		
Yes	614	9.9	64.3±23.0		
Type of diabetes					0.008
No diabetes	5574	90.1	67.3±22.0		
T1DM	131	2.1	63.7±25.1		
T2DM	483	7.8	64.4±22.5		

### Diabetes and the global health status (QL)

According to the American Joint Committee on Cancer (AJCC) Cancer Staging Manual, 7th Edition [21], tumor-node-metastasis (TNM) cancer staging was calculated using the indicators of tumor size (T), regional lymph node metastasis (N), and distant metastasis (M). The scoring conditions of the global health status (QL) were as follows: (1) if the tumor is larger, the scoring of QL is lower, (2) if the range of regional lymph node metastasis is larger, the scoring of QL is lower, and (3) if the distant metastasis is farther, the scoring of QL is lower.

As shown in Table 2 and Table 3, the size of the tumor, regional lymph node metastasis, and distant metastasis were significantly associated with decreased QL. Diabetes, either T1DM or T2DM or both, significantly reduced QL. This effect of diabetes on QL is independent of tumor size, regional lymph node metastasis, distant metastasis and TNM.

**Table 2:** The original scoring of QL on T, N, M and TNM staging. (Original scores, Mean ± SD).

	DM n = 614	T1DM n = 131	T2DM n = 483	No DM n = 5574	*P*_original_	*P*_original_	*P*_original_
					DM vs. No DM	T1DM vs. No DM	T2DM vs. No DM
Primary tumor diameter(T)							
T0	73.7±23.6	68.2±30.9	75.5±21.0	69.8±25.6	0.508	0.180	0.123
T1	65.1±24.3	64.2±25.3	65.4±24.0	67.8±21.6	0.009	0.039	0.061
T2	62.5±20.9	65.2±24.3	61.9±20.1	66.8±21.9	0.558	0.166	0.181
T3	61.2±20.4	65.3±8.2	60.6±21.7	64.1±23.9	0.149	0.020	0.388
T4	54.2±17.2	36.1±21.0	59.1±13.2	63.7±16.9	0.850	0.854	0.178
Regional lymph node metastasis(N)							
N0	65.6±21.9	65.4±24.2	65.7±21.3	67.9±21.9	0.623	0.160	0.212
N1	60.4±24.8	58.3±29.9	61.0±23.2	66.3±22.4	0.118	0.002	0.876
N2	64.5±29.7	75.0±20.4	60.1±31.4	64.4±20.8	0.000	0.982	0.000
N3	57.6±23.8	56.9±17.8	57.9±26.0	63.1±23.1	0.864	0.352	0.464
Distant metastasis(M)							
M0	64.5±22.9	64.0±25.3	64.7±22.3	67.8±21.9	0.255	0.006	0.938
M1	61.0±24.6	60.4±23.9	61.2±25.1	59.5±22.7	0.370	0.797	0.388
TNM staging							
Stage0	72.6±24.2	65.8±31.5	74.7±21.5	69.7±26.0	0.560	0.199	0.145
Stage1	66.7±23.2	66.3±24.3	66.8±23.0	68.2±21.5	0.144	0.586	0.267
Stage2	62.4±21.7	63.2±25.5	62.2±20.7	67.6±22.0	0.506	0.083	0.102
Stage3	58.6±23.3	52.8±23.2	59.8±23.4	65.8±21.4	0.309	0.608	0.352
Stage4	56.0±24.6	60.4±23.9	58.2±25.1	59.5±22.7	0.370	0.797	0.388

1. Primary tumor diameter (T): T0; ≤20mm(T1); 20mm<T≤50mm(T2); >50mm(T3); Tumor in any size with direct extension to chest wall and skin (T4). 2. Regional lymph node metastasis (N):N0,No regional lymph node metastasis;N1,Metastasis to movable ipsilateral axillary lymph node; N2, Metastasis to ipsilateral axillary lymph node that are fixed to one another or to other structures;N3, Metastasis to ipsilateral internal mammary lymph node. 3. Distant metastasis (M):M0, No distant metastasis; M1, Distant metastasis (Includes metastasis to ipsilateral supraclavicular lymph node). 4. TNM staing:Stage0(TisN0M0);Stage1(T1N0M0);Stage2(T0N1M0,T1N1M0,T2N0M0,T2N1M0,T3N0M0); Stage3(T0N2M0,T1N2M0,T2N2M0,T3N1M0,T3N2M0,T4N0M0,T4N1M0,T4N2M0,TiN3M0);Stage4(TiNiM1). 5. Original scores, Mean ± Standard Deviation

**Table 3:** The adjusted scoring of QL on T, N, M and TNM staging. (Adjusted scores, Mean ±SE).

	DM n = 614	T1DM n = 131	T2DM n = 483	No DM n = 5574	*P*_adjusted_DM vs. No DM	*P*_adjusted_ T1DM vs. No DM	*P*_adjusted_ T2DM vs. No DM
Primary tumor diameter(T)							
T0	74.5±4.6	71.7±8.3	77.0±4.9	71.1±1.8	0.493	0.339	0.256
T1	64.6±1.4	65.2±2.2	64.7±1.5	68.3±0.4	0.009	0.143	0.023
T2	62.4±1.7	64.6±3.9	61.9±1.8	67.0±0.6	0.009	0.531	0.008
T3	61.9±3.5	65.5±9.8	61.3±3.8	63.5±1.5	0.694	0.845	0.601
T4	52.3±4.9	42.3±0.2	56.0±5.7	64.1±1.8	0.031	0.043	0.180
Regional lymph node metastasis(N)							
N0	64.9±1.1	64.2±2.5	65.0±1.3	68.4±0.4	0.003	0.099	0.011
N1	60.7±2.3	58.6±4.6	61.5±2.7	66.5±0.7	0.018	0.093	0.071
N2	64.2±4.4	74.7±10.0	61.7±4.9	65.0±1.5	0.848	0.294	0.510
N3	58.2±4.9	58.6±9.3	58.6±5.7	61.9±1.7	0.476	0.721	0.583
Distant metastasis(M)							
M0	63.9±1.0	62.9±2.2	64.2±1.1	68.2±0.3	0.000	0.017	0.001
M1	61.9±3.6	62.5±7.9	61.9±4.0	59.8±1.3	0.569	0.746	0.622
TNM staging							
Stage0	72.7±5.1	45.0±15.3	76.3±5.4	71.1±2.0	0.764	0.108	0.324
Stage1	66.0±1.5	66.4±3.2	65.9±1.7	68.6±0.5	0.108	0.481	0.134
Stage2	62.1±1.5	62.6±3.3	62.1±1.7	67.9±0.5	0.000	0.088	0.001
Stage3	58.5±3.0	55.1±7.2	59.3±3.3	65.9±1.1	0.024	0.140	0.061
Stage4	61.9±3.6	62.5±7.9	61.9±4.1	59.8±1.3	0.569	0.746	0.622

1.Primary tumor diameter (T): T0; ≤20mm(T1); 20mm<T≤50mm(T2); >50mm(T3); Tumor in any size with direct extension to chest wall and skin (T4). 2.Regional lymph node metastasis(N):N0,No regional lymph node metastasis;N1,Metastasis to movable ipsilateral axillary lymph node; N2, Metastasis to ipsilateral axillary lymph node that are fixed to one another or to other structures;N3, Metastasis to ipsilateral internal mammary lymph node. 3. Distant metastasis (M):M0, No distant metastasis; M1, Distant metastasis (Includes metastasis to ipsilateral supraclavicular lymph node). 4. TNM staing:Stage0(TisN0M0);Stage1(T1N0M0);Stage2(T0N1M0,T1N1M0,T2N0M0,T2N1M0,T3N0M0); Stage3(T0N2M0,T1N2M0,T2N2M0,T3N1M0,T3N2M0,T4N0M0,T4N1M0,T4N2M0,TiN3M0);Stage4(TiNiM1) 5.Adjusted factors: Age, Marital status, BMI, Education, Personal income, Time after diagnosis, Treatment and Other chronic diseases (hypertension, hyperlipidemia, hyperuricemia, coronary heart disease, respiratory disease, stroke, musculoskeletal disease). 6. Adjusted scores, Mean ± Standard Error

### Diabetes and QOL for breast cancer patients

Table 4 presents the association between diabetes and QOL for breast cancer patients. After adjusting for social-demography, diagnosis and treatment of the tumor, tumor stage and other chronic diseases, diabetic patients got significantly lower EORTC QLQ-C30 scores in terms of functional dimensions, including physical functioning (PF), role functioning (RF), emotional functioning (EF) and global health status (QL) than those without diabetes. The scorings of diabetic patients in symptom dimensions, including fatigue (FA), nausea and vomiting (NV), pain (PA), dyspnoea (DY), insomnia (SL), constipation (CO) and diarrhoea (DI), as well as economic difficulties were all higher than the scorings of survivors without diabetes. The scorings of T1DM in functional dimensions were all lower than the scorings of survivors who do not have T1DM, in symptom dimensions except for AP and CO, and the scorings of T1DM patients in symptom dimension were all higher than the scorings of survivors without T1DM, and the scorings in CF, EF, QL, FA, NV, DY, SL, CO and DI all have statistical significance. The scorings of breast cancer patients who also have T2DM in functional dimension were all lower than the scorings of survivors who do not have T2DM, in symptom dimensions except for FA, CO and DI, and the scorings of T2DM patients in symptom dimension were all higher than the scorings of survivors who do not have T2DM, and the scorings in PF, RF, QL, FA, PA, DY, SL, AP, CO and FI all have statistical significance.

**Table 4:** The association between diabetes mellitus and original scores of QOL among breast cancer survivors. (Original scores, (Mean_DMi_-Mean _No DM_)(Mean_DMi_±SD)).

		DM,n = 614	T1DM,n = 131	T2DM,n = 483
		No DM,n = 5574	No DM,n = 5574	No DM,n = 5574
EORTC QLQ-C30				
Global health status	QL	-3.0(64.3±23.1) **	-1.3(65.7±24.1)	-2.8(64.4±22.5) * *
Physical functioning	PF	-2.6(82.4±14.9) ***	-2.4(82.4±15.2) *	-2.5(82.4±15.5) ***
Role functioning	RF	-1.3(89.3±19.1)	-1.1(89.4±18.7)	-1.2(89.4±18.4)
Cognitive functioning	CF	-1.8(79.5±18.7) *	-3.6(77.6±18.8) **	-0.7(80.5±17.8)
Emotional functioning	EF	-1.7(83.2±18.0) *	-3.3(81.5±19.7) **	-1.0(83.8±17.5)
Social functioning	SF	-1.4(80.9±21.9)	-2.3(79.9±23)	-1.7(80.6±22.6)
Fatigue	FA	2.2(26.9±18.8) **	2.2(27.0±19.2) *	1.6(26.4±18.0)
Nausea and vomiting	NV	0.8(3.5±10.8) *	1.8(4.5±11.8) **	-0.2(2.6±8.4)
Pain	PA	2.6(17.7±18.3) ***	3.7(18.9±18.8) **	2.0(17.3±18.1) **
Dyspnoea	DY	4.4(16.9±22.1) ***	4.5(17.3±21.6) ***	3.8(16.4±21.6) ***
Insomnia	SL	3.9(22.5±25.0) ***	4.2(23.1±24.8) **	3.1(21.9±25.9) **
Appetite loss	AP	1.0(7.6±15.7)	0.9(7.5±16.3)	0.9(7.6±16.5)
Constipation	CO	3.3(12.2±20.7) ***	0.7(9.9±19.4)	3.1(12.0±20.5) ***
Diarrhoea	DI	2.7(9.7±18.4) ***	4.0(11.1±18.6) ***	1.8(8.9±17.9) *
Financial difficulties	FI	3.8(31.0±32.3) **	4.7(32.1±32.5)	4.3(31.6±33.1) **
QLQ-BR23				
Body image	BRBI	-2.9(64.1±25.8) **	-2.7(64.1±25.5) *	-2.3(64.6±25.7) *
Sexual functioning	BRSEF	-2.9(95.0±13.0) ***	-2.1(94.3±13.1)	-3.4(95.4±12.1) ***
Sexual enjoyment	BRSEE	-4.4(95.1±13.6) ***	-3.5(94.6±13.2)	-4.9(95.7±12.5) ***
Future perspective	BRFU	-4.9(58.0±32.9) ***	-3.2(59.3±33.1)	-4.9(57.9±33.5) **
Side effects	BRST	3.7(19.4±13.1) ***	3.9(19.8±13.5) ***	3.4(19.2C13.4) ***
Breast symptoms	BRBS	4.5(20.8±20.8) ***	5.4(21.9±20.2) ***	3.9(20.3±20.8) ***
Arm symptoms	BRAS	3.6(25.5±22.2) ***	3.9(26.0±21.2) *	3.5(25.5±22.8) ***
Upset by hair loss	BRHL	3.3(21.5±27.3) **	2.7(21.1±25.9)	3.4(21.7±28.1) **

1. DM, contained T1DM and T2DM; 2.Original scores, (Mean_DMi_-Mean _No DM_)(Mean_DMi_ ±SD): The difference value between mean score of quality of life instrument with having diabetes and having no it. (mean score of quality of life instrument with having diabetes). 3.* *P* <0.05, ** *P* <0.01, *** *P* <0.001.

As shown in Table 5, the scorings of diabetic patients in two QLQ-BR23 functional dimensions, including body image (BRBI) and future perspective (BRFU) were significantly lower than the scorings of survivors without diabetes. The scorings of diabetic patients in 4 symptom dimensions, including side effects (BRST), breast symptoms (BRBS), arm symptoms (BRAS) and upset by hair loss (BRHL) were higher than the scorings of survivors without these 4 symptoms, and all the scorings have statistical significance. The scorings of breast cancer survivors combined with T1DM in functional dimensions BRSEF and BRSEE were all lower than related scorings of survivors without T1DM. The scorings of T1DM patients in symptom dimensions were all higher than the scorings of survivors without T1DM, and the scoring difference in BRST, BRBS and BRHL has statistical significance. Except for functional dimension BRBI, the scorings of breast cancer survivors combined with T2DM in other functional dimension were all lower than the scorings of survivors who do not have T2DM. The scorings of T2DM patients in symptom dimensions were all higher than the scorings of survivors without T2DM, and the scorings of symptom dimensions and functional dimensions all have statistical significance.

**Table 5:** The association between diabetes mellitus and adjusted scores of QOL among breast cancer survivors. (Adjusted scores,(Mean_DMi_-Mean _No DM_) (Mean_DMi_±SE))

		DM,n = 614	T1DM,n = 131	T2DM,n = 483
		(No DM,n = 5574)	(No DM,n = 5574)	(No DM,n = 5574)
EORTC QLQ-C30				
Global health status	QL	-2.1(65.2±1.1) **	-0.2(65.6±1.8) *	-3.3(67.4±1.3) *
Physical functioning	PF	-2.9(82.3±0.7) ***	-1.4(82.3±1.1)	-1.4(82.3±0.8) ***
Role functioning	RF	-1.8(89.0±0.9) *	-0.6(89.1±1.4)	-1.0(89.0±1.0) *
Cognitive functioning	CF	-2.0(79.2±0.9)	-2.6(78.4±1.5) *	-2.4(80.9±1.0)
Emotional functioning	EF	-2.9(82.0±0.9) **	-3.0(81.2±1.4)*	-0.4(82.9±1.0)
Social functioning	SF	-1.9(80.4±1.1)	-0.7(80.5±1.8)	-3.7(79.0±1.2)
Fatigue	FA	2.8(27.1±1.0) **	1.2(27.0±1.6) *	-1.0(25.9±1.1) *
Nausea and vomiting	NV	1.1(3.9±0.5) *	1.1(4.2±0.8) ***	1.0(2.6±0.5)
Pain	PA	2.3(17.5±0.9) **	0.9(17.4±1.4)	1.6(17.8±1.0) **
Dyspnoea	DY	5.0(17.3±1.0) ***	2.2(17.2±1.6) **	0.7(16.4±1.1) ***
Insomnia	SL	4.5(23.2±1.2) ***	3.2(23.7±1.9) *	0.2(22.2±1.3) **
Appetite loss	AP	1.0(7.7±0.8) **	-1.5(6.7±1.3)	0.6(7.8±0.9) **
Constipation	CO	1.4(10.4±0.9) **	-1.7(9.2±1.5) *	-1.7(9.2±1.0) **
Diarrhoea	DI	2.9(10.1±0.8) **	2.9(10.8±1.3)***	-1.9(8.5±0.9)
Financial difficulties	FI	4.7(31.5±1.5) **	0.5(30.6±2.4)	6.6(33.7±1.7) ***
QLQ-BR23				
Body image	BRBI	-3.0(64.1±1.3)**	0.3(65.0±2.1)	0.0(64.8±1.5) *
Sexual functioning	BRSEF	-0.8(93.1±0.7)	-1.0(92.4±1.2)	-2.9(94.3±0.8) *
Sexual enjoyment	BRSEE	-1.7(92.8±0.9)	-0.5(92.1±1.4)	-3.3(94.0±1.0)*
Future perspective	BRFU	-3.0(60.2±1.6) **	2.9(62.4±2.7)	-1.7(60.1±1.8) **
Side effects	BRST	4.0(20.0±0.7) ***	2.6(20.3±1.1)**	1.6(19.8±0.7) ***
Breast symptoms	BRBS	5.3(21.6±0.9) ***	3.3(21.9±1.4)***	0.6(20.6±1.0) ***
Arm symptoms	BRAS	3.7(25.6±1.0) **	2.3(25.8±1.7)	2.5(25.9±1.2) **
Upset by hair loss	BRHL	4.3(22.0±1.3) **	3.1(22.5±2.1)	1.7(21.7±1.5) *

Notes: 1. DM, contained T1DM and T2DM; 2.Adjusted factors: Age, Marital status, BMI, Education, Personal income, Time after diagnosis, Treatment, Tumor staging and Other chronic disease (hypertension, hyperlipidemia, hyperuricemia, coronary heart disease, respiratory disease, stroke, musculoskeletal disease). 3.Adjusted scores,(Mean_DMi_-Mean _No DM_)(Mean_DMi_±SE): The difference value between mean score of quality of life instrument with having diabetes and having no it. (mean score of quality of life instrument with having diabetes). 4. * *P* <0.05, ** *P* <0.01, *** *P* <0.001.

## Discussion

WHO highlights that comprehensive measures are needed to improve the QOL of chronic disease patients [22]. Diabetes and breast cancer can not only threaten the health and mortality rate of patients, but also affects the QOL of patients [23]. Survival alone is not sufficient for breast cancer survivors, who also want to live a life without health concerns. QOL has also been shown to be a significant prognostic factor for mortality and cancer recurrence [24, 25]. In the current investigation, we carried out a relatively large cross-sectional study to explore the association between comorbidity of diabetes mellitus and QOL of breast cancer survivors. What was found in this study indicated that the comorbidity of diabetes significantly affects almost all dimensions of QOL of breast cancer survivors. Therefore, control and treatment of diabetes (especially T2DM) may improve the QOL of breast cancer survivors, including their general health condition and standard of life.

Diabetes may decrease cancer patients’ QOL in various ways. Diabetes could promote more rapid growth of tumors through metabolic factors such as hyperglycemia, insulin resistance, and hyperinsulinemia [26–28]. Hyperinsulinemia in T2DM induced the expression and increased the binding capacity of Estrogen Receptor (ER) [29]. Diabetes is also an adverse factor that can affect the prognostic life quality of breast cancer patients [30]. Diabetes may also exert indirect effects on breast cancer because of the associated end-organ damage that may affect screening and treatment options, enhance treatment toxicity, and lead to worse outcomes [31].

The participants of this study came from Cancer Recovery Clubs in 34 cities all over China, and they may have good representativeness of the breast cancer survivors in China. It should be kept in mind that severe patients were not included, as well as the dead registered members after tumor diagnosis. Information on the disease was collected by self-reports, and some recall bias may exist.

Despite the fact that a strong association between comorbidity of diabetes and QOL of breast cancer survivors was found in our study, due to the inherent limitation of cross-sectional study design, we cannot determine specific causal relationships. There are the studies that indicate the treatment for diabetes, for instance, the long-acting insulin analog glargine may be responsible for the association with the risk of cancer [32, 33]. Meanwhile, the chemotherapy for the treatment of breast cancer causes the increase of blood glucose [10]. In addition, we need to collect more clinical data (including prospective cohort studies of mass samples) and molecular biological evidence (for example, the test of insulin-like growth factor receptor in breast cancer samples) [34]. Further investigations are warranted to explore the mechanism of this effect.

## Conclusion

This study provides strong evidence that comorbidity of diabetes, especially T2DM, aggravates the QOL of breast cancer survivors. Thus, the prevention and proper treatment of comorbidity of diabetes are required to improve the QOL of diabetes and breast cancer survivors.

## Supporting Information

S1 Data Raw data of the manuscript. Raw data of the cross sectinal study on the associations between diabetes and quality of life among breast cancer survivors.
(XLSX)
